# Durable Red Blood Cell Transfusion Independence in a Patient with an MDS/MPN Overlap Syndrome Following Discontinuation of Iron Chelation Therapy

**DOI:** 10.1155/2015/253294

**Published:** 2015-03-30

**Authors:** Harpreet Kochhar, Chantal S. Leger, Heather A. Leitch

**Affiliations:** ^1^Department of Medicine, University of St. Eustatius, Tucker, GA 30084, USA; ^2^Division of Hematology, St. Paul's Hospital, University of British Columbia, Vancouver, BC, Canada V6Z 2A5

## Abstract

*Background*. Hematologic improvement (HI) occurs in some patients with acquired anemias and transfusional iron overload receiving iron chelation therapy (ICT) but there is little information on transfusion status after stopping chelation. *Case Report*. A patient with low IPSS risk RARS-T evolved to myelofibrosis developed a regular red blood cell (RBC) transfusion requirement. There was no response to a six-month course of study medication or to erythropoietin for three months. At 27 months of transfusion dependence, she started deferasirox and within 6 weeks became RBC transfusion independent, with the hemoglobin normalizing by 10 weeks of chelation. After 12 months of chelation, deferasirox was stopped; she remains RBC transfusion independent with a normal hemoglobin 17 months later. We report the patient's course in detail and review the literature on HI with chelation. *Discussion*. There are reports of transfusion independence with ICT, but that transfusion independence may be sustained long term after stopping chelation deserves emphasis. This observation suggests that reduction of iron overload may have a lasting favorable effect on bone marrow failure in at least some patients with acquired anemias.

## 1. Introduction

The myelodysplastic syndromes (MDS) are a heterogeneous group of clonal hematopoietic stem cell disorders characterized by ineffective blood cell production and a propensity to progress to acute myelogenous leukemia (AML). Survival and AML risk are predicted by the International Prognostic Scoring System (IPSS) and other prognostic scores [1–5]. Anemia is a common manifestation of MDS and most patients eventually become red blood cell (RBC) transfusion dependent (TD), which can have a significant adverse impact on quality of life [6]. Transfusion dependence in MDS has been associated with inferior overall survival (OS) and leukemia free survival (LFS), as has iron overload as measured by serum ferritin (SF) level [7–12]. Several analyses suggest that patients with lower risk MDS who received iron chelation therapy (ICT) had improved OS and possibly LFS compared to those who did not [13–21]. Some analyses suggest that the degree of survival benefit is associated with a longer period of chelation or with more effective reduction in SF [15, 20]. Similar data in smaller numbers suggest that patients with myeloproliferative neoplasms (MPN) and transfusional iron overload may also have inferior survival [22, 23] and might experience a survival benefit with chelation [24].

There are reports of hematologic improvement (HI) [25, 26] in a proportion of MDS [27–37] and MPN [38–44] patients receiving chelation, with some patients achieving transfusion independence (TI); however, information on transfusion status after stopping chelation is minimal and has received little attention to date. We report a 63-year-old woman with IPSS low risk refractory anemia with ring sideroblasts and thrombocytosis (RARS-T) who progressed to transfusion dependent myelofibrosis. Six weeks following the initiation of ICT, she became transfusion independent. When ICT was discontinued due to lack of financial coverage, the hemoglobin remained normal with ongoing transfusion independence seventeen months later.

This paper was written in accordance with the requirements of the Providence Research Institute Research Ethics Board.

## 2. Case Report

A 63-year-old woman was referred in October 2009 for a ten-month history of macrocytic anemia and thrombocytosis. She was otherwise asymptomatic. Physical examination was unremarkable. Blood work showed a white blood cell count of 11.5 × 10^9^/L, hemoglobin (Hb) 109 g/L, MCV 110 fL, platelets (PLT) 1,384 × 10^9^/L, neutrophils 6.3 × 10^9^/L, and SF of 223 ng/mL. Peripheral blood morphology showed thrombocytosis with large platelet forms. A bone marrow aspirate and biopsy showed RARS-T, an MDS/MPN-unclassifiable in the World Health Organization (WHO) provisional category [46]. Cytogenetic analysis showed a normal female karyotype and she was IPSS for MDS low risk with a predicted median survival of 5.7 years. Analysis for both JAK-2 (V617F) and BCR-ABL were negative.

In February 2010 she was started on cytoreduction with hydroxyurea because of thrombocytosis (Hb was 97 g/L and PLT count was 2,497 × 10^9^/L) associated with pseudohyperkalemia. In April 2010 she presented with fatigue; at that time the Hb was 76 g/L and the PLT count was 1,976 × 10^9^/L. In May 2010 the patient was started on RBC transfusions. By October 2010 the transfusion requirement (TR) was 2-unit (U) RBC every four weeks and the SF was 318 ng/mL with a transferrin saturation of 67%. There was no clinical bleeding throughout the patient's course. To rule out occult blood loss, the patient underwent CT colonography in November 2010, which was negative for any colonic lesions.

In March 2011 a leukoerythroblastic picture was noted on the blood smear. A repeat bone marrow biopsy showed clusters of megakaryocytes, myeloid proliferation, grade 3 reticulin fibrosis, and reduced erythropoiesis; the SF was 960 ng/mL. A diagnosis of myelofibrosis (MF) was made. Analysis for the JAK-2 V617F mutation remained negative. The Dynamic International Prognostic Scoring System-Plus (DIPSS+) score for primary myelofibrosis was 3, predicting a median OS of 35 months and 12% risk of progression to AML at ten years [47]. According to the MDS/fibrosis score [48] the projected median OS was 24 months with a three-year AML risk of 40%.

In May 2011, the SF was 1066 ng/mL. The DIPSS+/SF score at the onset of a SF ≥ 1000 predicts a median OS of 34 months [23]. She was assessed for a clinical trial of pomalidomide (versus placebo) and enrolled on the study in July 2011 after stopping cytoreduction. She was withdrawn from the study in January 2012 for lack of response and restarted hydroxyurea. The SF was 1970 ng/mL. In February 2012, the RBC TR was 2.5 units per 4 weeks. Fecal occult bloods were done in April 2012, which were negative. EPO was initiated in May 2012 at a dose of 40,000 U/week. The pretransfusion Hb prior to starting EPO were 81 g/L and six weeks later was 76 g/L.

Deferasirox (DFX) was started in June 2012 at a dose of 20 mg/kg/day and the patient received a last transfusion on August 7, 2012, at which time the pretransfusion Hb was 101 g/L and the SF was 1251 ng/mL. Two months later, the Hb was 133 (lower limit of normal 120) g/L and the SF was 911 ng/mL. At this point, the DIPSS+ score was low risk as she was transfusion independent, with a predicted median OS of 185 months [47]. The hydroxyurea dose was reduced as the PLT count was 237 (normal range 150–400) × 10^9^/L and EPO was stopped in January 2013.

In May 2013, the Hb remained normal at 125 g/L and the SF was 685 ng/mL. Deferasirox was stopped as she no longer met BC Pharmacare criteria for coverage, as she was no longer transfusion dependent. The Hb was 140 g/L and SF was 541 ng/mL in December 2013. In October 2014, 60 months from diagnosis of RARS-T, 41 months from diagnosis of MF, 43 months from the onset of transfusion dependence, 26 months from achieving transfusion independence, 33 months after stopping study medication, 21 months after stopping EPO, and 17 months after stopping deferasirox the Hb remained normal at 127 g/L and SF was 493 ng/mL.

**Figure 1:**
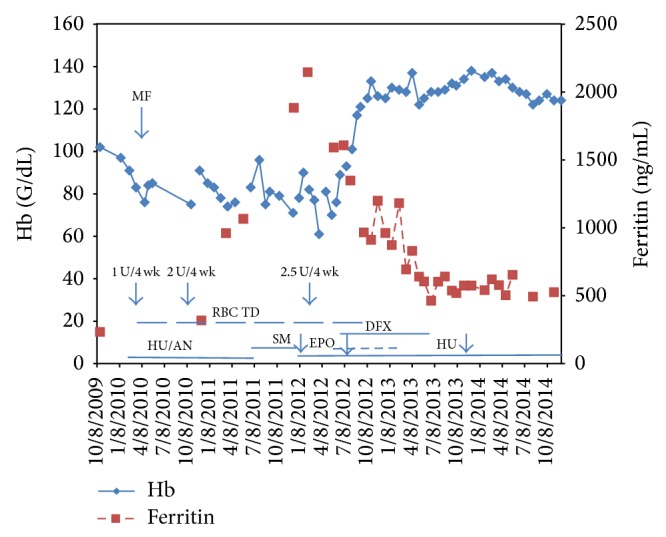
Clinical course of a patient with RARS-T evolved to myelofibrosis. Red blood cell transfusion dependence (RBC TD) started in 2010 and ended in August 2012, with number of RBC units (U) per 4 weeks (wk) indicated by the arrows. Hemoglobin (Hb) levels are pretransfusion measurements during the period of RBC TD and are otherwise regular measurements. Hydroxyurea (HU) and/or anagrelide (AN) were given from February 2010 to June 2011 with multiple dose adjustments to keep the platelet count less than 1000 × 10^9^/L. These were stopped as a requirement of a clinical trial of pomalidomide versus placebo (study medication indicated by SM); SM was given from July 2011 to January 2012 and stopped for lack of response. HU was resumed at doses of 2, 1.5, and 1 g/day, respectively, as indicated by the arrows (left to right), to keep the platelet count less than 1000, 400, and 400 × 10^9^/L, respectively. Erythropoietin (EPO) was started in May 2012 at 40,000 U/wk and stopped in January 2013 because of a Hb of 130 g/dL. Deferasirox (DFX) was started in June 2012 (5 weeks after EPO) at a dose of 1500 mg/day (20 mg/kg/day). The patient's last RBC transfusion was on August 7, 2013.

Cytoreduction for thrombocytosis was given from February 2010 to June 2011 and was hydroxyurea and/or anagrelide with multiple dose adjustments to keep the PLT count <1000 × 10^9^/L while minimizing transfusion requirements. Cytoreduction was stopped as a requirement of the clinical trial. Hydroxyurea was resumed in January 2012 at a dose of 2 g/day and then decreased to 1.5 and 1 g in July 2012 and November 2013, respectively, to keep the platelet count less than 1000, 400, and 400 × 10^9^/L, respectively. She is currently receiving hydroxyurea 500 mg once daily and the platelet count is 339 × 10^9^/L. In February 2015, a third analysis for the JAK-2 V617F mutation remained negative. The Hb, SF levels, transfusion requirements, and medications received over the patient's course are shown in Figure 1.

## 3. Literature Review

Characteristics of MDS patients reported to have achieved sustained TI after stopping ICT are summarized in Table 1. The median age at diagnosis of MDS or MPN of the seven reported cases was 61 (range 18–67) years, and five patients received deferoxamine (DFO) with two receiving DFX. The median duration of ICT was 20 (12–30) months and median time to RBC TI 20 (1.5–62) months (36 months for DFO and 3.25 months for DFX). The duration of RBC TI ranged from 3 to 36 months (five cases were reported as a range only).

## 4. Discussion

Transfusion independence following the initiation of iron chelation therapy, and while continuing to receive ICT, has been reported in both MDS and MPN. In larger series of lower IPSS risk MDS, erythroid response rates varied from 11% [36] to 45.6% [37]. A 12% rate of transfusion independence was seen in one study, and the probability of TI after adjusting for death and MDS progression was 2.6, 12.3, and 15.5 at 6, 9, and 12 months, respectively [35]. In a study of 23 patients with MPN receiving deferasirox, 18 were evaluable for response. A persistent increase in Hb of greater than 1.5 g/dL was observed in 5 patients, with 3 others becoming TI for an overall HI rate of 44% and TI rate of 17% [44]. Similarly, in 561 transfusion dependent patients with MF, 103 of whom received chelation, significantly lower rates of thrombocytopenia, pancytopenia, and emergency room visits were reported, adjusted incidence rate ratio of 0.54, 0.53, and 0.77, respectively, *P* < 0.0001 for all [43].

The mechanism of HI in MDS and MPN patients receiving chelation is a matter of active investigation. Though there appeared to be a greater reduction in serum ferritin levels in MDS patients with HI compared to those without, this difference did not reach statistical significance [31]. However, hematologic improvement has been reported with deferasirox [27, 31–33, 35–37, 41, 49–54], deferoxamine [28–30, 37], and deferiprone [40], suggesting a class effect associated with iron reduction. This is supported by the observation of Jensen et al. that HI following deferoxamine was associated with greater reduction in liver iron concentration as measured by MRI [29]. Time to HI was a median of three (range 1–15) months with deferasirox and nine months with deferoxamine in one study [37], and TI was achieved after receiving deferoxamine for 18 to 26 months in another [29]. Whether HI is a result of reduction in organ and total body iron or from modulating other processes associated with iron overload remains to be clarified.

Mechanisms of HI with chelation that have been suggested include repression of the mTOR pathway, which reduced myeloid leukemia tumor volume in a preclinical model [55]. Deferasirox inhibits signaling via the nuclear transcription factor NF*κ*B; however this effect was not observed with deferoxamine or deferiprone, so it does not account for the HI observed with all three chelators [56]. In one study, suppression by deferasirox of helper T-type 1 cells and T regulatory cells was seen along with a shift toward a helper T-cell type 2 phenotype, indicating that alterations in immune surveillance may occur [57]. Also suggested are the following: a direct effect on the neoplastic clone or the bone marrow environment; promotion of iron release from stores allowing use by hemopoietic tissue; and an increase in endogenous EPO levels [58]. An attractive model is a reduction by chelation in oxidative stress, which is induced in states of iron overload by virtue of the ability of iron to transfer electrons, resulting in the formation of reactive oxygen species (ROS) [59]. In preclinical models, increased ferritin levels are associated with a reduction in erythroid colony formation in vitro [60, 61]; iron overload induces apoptosis in erythroid precursors, and this correlates with the presence of ROS in CD34+ cells [62]. Similarly, measures of oxidative DNA damage are increased with transfusional iron overload and reduced following chelation with deferasirox for a period of three months; interestingly, this effect preceded an observed reduction in elevated serum ferritin levels [63]. Reductions in labile plasma iron (LPI), which is redox reactive, with chelation with deferasirox were demonstrated in two clinical studies [31, 32]. Both showed a reduction in LPI into the normal range, in the US03 study by three months and in the EPIC trial by postadministration at baseline. In another analysis, intracellular ROS within erythrocytes was significantly suppressed following three months of chelation (*P* < 0.05) [64]. Taken together, demonstrated effects of oxidative stress on lipids, proteins, and nucleic acids [55, 63] could account for an increase in apoptosis observed with iron overload [61, 62] and relief of this could lead to HI and possibly to a survival benefit observed with chelation in noncontrolled analyses. To date, however, oxidative stress has not been definitively tied to clinical endpoints, possibly because it is reduced too quickly by chelation to accurately capture an association [31]. Measures of the longer lasting cellular results of oxidative stress might in future prove more revealing [63].

There has been little emphasis in the literature on outcomes in patients with marrow failure syndromes after stopping chelation following HI. In a case report of HI with deferasirox (DFX) in primary myelofibrosis (PMF), interruption of chelation at one month resulted in loss of HI which was regained when chelation was resumed [41]. A second patient with PMF attained TI after only 4 weeks of chelation with DFX, which was interrupted at 8 weeks for a decline in ferritin level. Following another 8 weeks, DFX was resumed for an increase in ferritin level; the patient remained TI for 6 months at the time of publication, including the 8 weeks off chelation [65]. In a third case, a PMF patient became TI with chelation after five months; deferasirox was stopped following twelve months of chelation, and TI was maintained two years later [38]. In a report of seven MDS patients with HI following deferoxamine, TI was not the focus of the study; however five patients became TI after treatment for 18 to 26 months. The duration of TI in this study was a minimum of 3 months and up to 36 months at the time of publication. Our patient received twelve months of chelation before it was stopped and durable HI was observed. Angelucci et al. [35] demonstrated a time-dependence in TI rates during the first 12 months of chelation with DFX, but the optimal duration of chelation to maximize the incidence of TI remains to be clarified. Also unclear topics for future investigation are as follows: which characteristics of MDS and MPN patients predict HI with chelation; and which MDS and MPN subtypes are more likely to respond.

We cannot rule out a clonal switch in our patient, as no follow-up marrow was done, and analysis for the JAK2 V617F mutation remained negative throughout her course. However, clonal evolution generally results in worsening of the hematologic picture, and there are hints of this occurring in our patient before treatment with deferasirox, as indicated by the increasing transfusion requirement and less exuberantly increased platelet count, which may indicate progression of fibrosis beyond the proliferative phase. In the Jensen study [29], it appeared that patients received supportive care alone beyond ICT. The PMF patient reported by di Tucci et al. [38] received no other treatment beyond ICT. Our patient, though she received EPO, did not respond to EPO alone and maintained HI and TI 21 months after EPO was stopped. Though HI with EPO would not be surprising, to our knowledge, sustained TI long term after stopping EPO has not been reported. We also cannot rule out a delayed response to study medication, presuming she was randomized to receive pomalidomide, though this would have occurred more than a year from starting, and more than 6 months from stopping, and response is ongoing over 3.5 and 3 years from starting and stopping. In trials of pomalidomide for myelofibrosis, the reported median time to response was 1.6 months and response duration was 6.7 months, with one patient with transfusion independence having a response of 15 months [66]. In a second study, the median anemia response duration was 16 months, and in a third the range was 3.2–16.9 months, all considerably shorter than the response duration of our patient [67, 68]. To our knowledge, there is no information available as to expected response of thrombocytosis to pomalidomide, nor is there specific information on pomalidomide activity in RARS-T. There is, however, a case report of RARS-T treated with lenalidomide which resulted in resolution of splenomegaly but severe and prolonged pancytopenia [69]. EPO alone, while expected to improve RBC transfusion requirements in some patients, would not be expected to improve thrombocytosis, as was seen in this patient. An improvement in thrombocytosis was also observed in a PMF patient achieving TI following DFX [65]. Taken together, these observations indicate that some patients may achieve durable TI with ICT alone. Whether the HI is additive or synergistic with other therapies expected to induce HI remains to be defined [70].

In summary, our patient's achievement of durable TI even following discontinuation of iron chelation therapy improved prognostic scores predicting overall survival. In two other reports of this phenomenon, sustained TI was not a focus of one and potential mechanisms of HI were not discussed at any length [29, 38]. The current report highlights that durable transfusion independence may be achieved in some patients with acquired anemias following reduction of iron overload, suggesting a favorable impact on bone marrow failure in some patients with acquired anemias. The course of these patients may inform future analyses and clinical trial design.

## Figures and Tables

**Table 1:** Characteristics of patients reported to have achieved sustained transfusion independence after stopping iron chelation therapy.

Case	Age	FAB or WHO diagnosis	Karyotype at diagnosis	Risk score	ICT	Duration of ICT	Time to TI (mo.)	Duration of TI (mo.)	Duration of TI after stopping ICT (mo.)	Other treatment	Reference
1	67	RA	del(11)(q22q24)	NR	DFO^1^	30	52	NR	3–36^2^	None reported	[29]

2	46	RA	Normal	NR	DFO	25	20	24+	3–36^2^	None reported	[29]

3	41	RA	Normal	NR	DFO	20	36	20+	3–36^2^	None reported	[29]

4	18	RAEB	Normal	NR	DFO	20	20	6+	3–36^2^	None reported	[29]

5	65	MDS	+8	NR	DFO	15	50	NR	3–36^2^	Hydrea^3^	[29]

6	61	PMF	NR	Int.^4^	DFX	20	5	36+	22+	None	[38]

7	63	RARS-T	Normal	Low^5^	DFX	12	1.5	22	12+	EPO^6^	Current report

AML, acute myelogenous leukemia; DFO, deferoxamine; DFX, deferasirox; Int., intermediate; FAB, French American British; hydrea, hydroxyurea; IPSS, International Prognostic Scoring System; ICT, iron chelation therapy; MDS, myelodysplastic syndrome; mo., months; MPN, myeloproliferative neoplasm; PMF, primary myelofibrosis; RA, refractory anemia; RAEB, refractory anemia with excess blasts; MDS, myelodysplastic syndromes; MPN, myeloproliferative neoplasm; NR, not reported; PMF, primary myelofibrosis; RARS-T, refractory anemia with ring sideroblasts and thrombocytosis; TI, transfusion independence; WHO, World Health Organization; +, indicates ongoing TI.

^1^TI achieved 15 months after chelation was stopped. ^2^Duration of TI after stopping chelation for cases 1–5 was reported as a range only. ^3^Hydroxyurea was given after stopping chelation due to progression to AML. ^4^Dupriez score (Dupriez et al., 1996 [45]). ^5^IPSS score for MDS (Greenberg et al., 1997 [1]). ^6^No response to EPO.
